# Effects of dysfunctional beliefs about sleep on sleep quality and mental health among patients with COVID-19 treated in Fangcang shelter hospitals

**DOI:** 10.3389/fpubh.2023.1129322

**Published:** 2023-02-23

**Authors:** Jiaxi Peng, Tian Zhang, Yijun Li, Lin Wu, Xiyuan Peng, Chenxi Li, Xinxin Lin, Jing Yu, Li Mao, Jingjing Sun, Peng Fang

**Affiliations:** ^1^Mental Health Education Center & College of Teachers, Chengdu University, Chengdu, China; ^2^Department of Military Medical Psychology, Air Force Medical University, Xi'an, China; ^3^Department of Radiology, 986 Hospital of Air Force Medical University, Xi'an, China; ^4^No. 10 Outpatient Department of Internal Medicine, 986 Hospital of Air Force Medical University, Xi'an, China; ^5^School of Biomedical Engineering, Air Force Medical University, Xi'an, China; ^6^Shaanxi Provincial Key Laboratory of Bioelectromagnetic Detection and Intelligent Perception, Xi'an, China

**Keywords:** Fangcang shelter hospitals, COVID-19, anxiety, depression, insomnia, dysfunctional beliefs about sleep

## Abstract

**Introduction:**

With the COVID-19 pandemic in China, a large number of mild or ordinary confirmed cases have been sent to Fangcang shelter hospitals for treatment. We aimed to investigate the mental health condition of Fangcang patients 2 years after the pandemic when patients knew more about COVID-19 and the virus was less virulent. We focused on the effect of dysfunctional beliefs and attitudes about sleep on depression, anxiety, and insomnia.

**Methods:**

A total of 1,014 patients from two large Fangcang shelter hospitals in Shanghai between 22 April and 8 May 2022 completed a set of questionnaires comprising: the Dysfunctional Beliefs and Attitudes about Sleep scale, the Generalized Anxiety Disorder scale, the Patient Health Questionnaire, and the Insomnia Severity Index scale.

**Results:**

Results show that the positive screening rates for anxiety, depression, and insomnia among tested patients were 55.3, 27.0, and 47.8%, respectively. Patients were more likely to report higher anxiety, depression, and insomnia, and to endorse affective and sleep disorders if they were: female, aged 18–40 years, with undergraduate course or above, white-collar employees, or those who thought the pandemic would have severe economic effects. About 51.4% of the participants had dysfunctional beliefs about sleep to varying degrees. Compared with patients who had accurate beliefs about sleep, the ratios of insomnia, anxiety, and depression were significantly higher among patients with dysfunctional beliefs about sleep.

**Discussion:**

Attention should be paid to the mental health problems of patients in Fangcang shelter hospitals. The results indicate that dysfunctional beliefs about sleep significantly increased anxiety, depression, and insomnia of Fangcang patients.

## Introduction

With the COVID-19 pandemic in China, a large number of mild or ordinary confirmed cases have been sent to Fangcang shelter hospitals for treatment. Fangcang shelter hospitals are rapidly-deployable, temporary hospitals that integrate basic medical services and life safeguarding functions, and can be reconstructed from the existing large architecture facilities in cities, such as gymnasia, exhibition centers, storage warehouses, or large workshops (1, 2). Despite the great contributions to preventing pandemic spread, patients in Fangcang shelter hospitals have suffered depression, anxiety, insomnia, and other psychological problems (3–6). For example, Dai et al. found the positive screening rates for anxiety and depression among patients in Fangcang shelter hospitals were 18.6, and 13.4%, respectively (3). Zhang et al. reported 49.6% of participants had depressive or anxiety symptoms, and the symptoms of both depression and anxiety were highly correlated with the degree of insomnia (6). Gu et al. found 25.2, 50.1, 54.4, 10.2, and 39.7% of patients in Fangcang shelter hospitals reported symptoms of post-traumatic stress, anxiety, depression, insomnia, and perceived stress, respectively (5). So far, however, there are relatively few studies on the mental health of patients in Fangcang shelter hospitals, and the existing relevant research focuses on the effects of demographic variables on the mental health index. There has been no deep discussion into the emotional and individual psychological variables related to sleep disorders among patients in Fangcang shelter hospitals.

Insomnia is one of the main psychological problems caused by the COVID-19 pandemic (7, 8). A meta-analysis showed 35.7% of the general population suffered various sleep problems amid the pandemic, and it went up to 74.8% among COVID-19 patients (9). Stanton et al. found that experience of sleep disorders during the COVID-19 pandemic was highly correlated with anxiety, depression, and stress (10). Lin et al. reported pregnant women easily suffered anxiety and depression during the COVID-19 pandemic, and these were both significantly correlated with their sleeping status (11). Similarly, Zhang et al. found the sleep disorders of patients in Fangcang shelter hospitals were significantly correlated with mental health (6). Thus, in this study, we aimed to discuss the individual factors affecting the mental health of patients in Fangcang shelter hospitals, starting from the factors influencing sleep.

Many empirical studies suggest that improper pre-sleep cognition and views, and worry or fear of probable insomnia may critically affect the occurrence, development, maintenance, and treatment of insomnia (12). These cognitive factors are collectively called “dysfunctional beliefs about sleep” (13). Reportedly, dysfunctional beliefs about sleep can significantly predict insomnia, stress, depression, anxiety, and suicidal ideation (14). Recent sleep quality studies amid the COVID-19 pandemic have shown that individuals' dysfunctional beliefs about sleep are significantly correlated with sleep quality and mental health indicators (15, 16). For instance, Sella et al. found changes in self-reported sleep quality were largely associated with changes in dysfunctional sleep-related beliefs in older adults during COVID-19 lockdowns. Reportedly, 18.6% of Italian adults suffered clinical insomnia, and the severity of insomnia was significantly correlated with dysfunctional beliefs about sleep (17). Idrissi et al. found that 82.3% of people suffered dysfunctional beliefs about sleep to different extents amid the pandemic, and these beliefs were significantly correlated with insomnia, anxiety, and depression (15).

The wave of the COVID-19 pandemic dominated by the Omicron variant started to spread in Shanghai in April 2022. At that point, about 110 Fangcang shelter hospitals are founded in Shanghai, with up to 250,000 beds; these mainly accepted and isolated patients who were asymptomatic or had mild infections (18). This study was targeted at patients treated in some Fangcang shelter hospitals in Shanghai during this period. The insomnia, depression, and anxiety of patients were investigated, and our study was primarily focused on how dysfunctional beliefs about sleep were related with sleep disorders and affective disorders. Compared with previous studies on the mental health of patients in Fangcang shelter hospitals, our data were collected 2 years after the pandemic when patients already knew more about COVID-19. Moreover, the Omicron variant was less virulent than previous variants (19). For these reasons, patients treated during the study period may have had better mental health than patients at early stages of the pandemic. Additionally, previous studies ignored the effects of individual cognitive factors on insomnia and affective disorders. Although the objective realities of Fangcang shelter hospitals—poor living facilities, 24-h lighting, limited personal space, concern about illness status—are major causes for the mental problems of patients, we believe the traits and cognition of patients also play important roles. This is the first study to consider the effects of dysfunctional beliefs about sleep on the insomnia, depression, and anxiety of patients in Fangcang shelter hospitals.

## Methods

### Participants

Convenience sampling was used to collect data from two large Fangcang shelter hospitals in Shanghai from 22 April to 8 May 2022. The inclusion criteria were: positive COVID-19 status, diagnosed as asymptomatic or mild patients; admission within the previous 2 weeks; age 16–65 years; breathing rate < 30 beats per minute; demonstration of cognizance and self-caring abilities in answering questionnaires; no history of self-reported psychopathy; no use of anti-depression or anti-anxiety drugs within one year; no other chronic diseases (e.g., hypertension, coronary heart diseases). With traditional written tests, some trained nurses informed the patients about the significance of this questionnaire. After the patients signed informed consent forms, they voluntarily participated in the questionnaire.

In total, 1,124 copies of the questionnaire were sent out, and 1,087 copies were returned. Of them, 73 copies were excluded due to their incompleteness. Finally, 1,014 copies were included in the analysis.

## Measures

### Demographic information

The demographic variables collected included gender (male or female); age (<18 years, 18–40 years, 40–60 years, >60 years); whether the individual was very worried about the economy (yes or no); education level (senior high school and below, technical school and junior college, undergraduate and above); and occupation (industrial workers, white-collar employees, students, unemployed and others).

### Dysfunctional beliefs and attitudes about sleep-16

The DBAS-16, developed by Morin, Vallières, and Ivers, consists of sixteen items that estimate the respondents' beliefs and attitudes about sleep (13). Some example items are “My sleep is unpredictable” and “Insomnia is destroying my life”. The DBAS-16 assesses the following four dimensions: consequences of insomnia, worry about sleep, sleep expectation, and prescription of drugs. The DBAS-16 was translated into Chinese and showed good reliability and validity (20). The responses were made using a 5-point Likert scale, ranging from 1 (*Strongly disagree)* to 5 (*Strongly agree)*. The summations of all items, and of the items belonging to each dimension, were calculated as the global score and score of each dimension, respectively. The global score ranged from 16 to 80. According to Fu, Ou, and Lu, a total score ≥ 48 indicates false beliefs about sleep, whereas scores lower than 48 indicate correct, or accurate, beliefs about sleep (20). The Cronbach's alpha coefficient of the DBAS-16 was 0.89 in the current study.

### Generalized anxiety disorder scale

GAD-7, which consists of seven items, was used to estimate the respondents' anxiety (21). Some example items are “I worry too much about different things” and “I become easily annoyed or irritable”. The responses were made using a 4-point Likert scale, ranging from 0 (*Not at all)* to 3 (*Nearly every day)*. The summation of all items was calculated for the global score, ranging from 0 to 21, which indicated severity of anxiety symptoms: minimal (0–4), mild (5–9), moderate or severe (10–22). The Cronbach's alpha coefficient of GAD-7 was 0.95 in the current study.

### Patient health questionnaire-9

PHQ-9, which consists of nine items, was used to estimate patients' depressive symptoms (23). Some example items were “I experience poor appetite or overeating” and “I feel tired or have little energy”. The responses were made using a 4-point Likert scale, ranging from 0 (*Not at all)* to 3 (*Nearly every day)*. The summation of all items was calculated as the global score, ranging from 0 to 27, which indicated the severity of depression symptoms: minimal (0–9), mild (10–14), moderate or severe (6, 15–27). The Cronbach's alpha coefficient of PHQ-9 was 0.92 in the current study.

### The insomnia severity index

The ISI, which consists of five items, was used to estimate the severity of insomnia (24). Some example items were “I have difficulty falling asleep” and “I have difficulty staying asleep”. The responses were made using a 5-point Likert scale, ranging from 0 (*None)* to 4 (*Very)*. The summation of all items was calculated as the global score, ranging from 0 to 28, which indicated severity of insomnia: minimal (0–7), mild (8–14), moderate or severe (15–28). The Cronbach's alpha coefficient of PHQ-9 was 0.83 in the current study.

### Data analysis

Statistical analyses were carried out using SPSS 22.0. Descriptive statistics, correlation analysis, and logistic regression analysis were used to analyze the data collected.

## Results

Table 1 lists the demographic information of the participants, and the mental health differences observed in terms of gender, age, education level, economic concern, and occupation. Results showed depression (*F* = 12.39, *P* < 0.01), anxiety (*F* = 12.94, *P* < 0.01), and insomnia (*F* = 6.08, *P* = 0.02) were more severe among females than males. Depression (*F* = 12.39, *P* < 0.01), anxiety (*F* = 12.94, *P* < 0.01), and insomnia (*F* = 6.08, *P* = 0.02) were significantly different among age groups, and the scores were highest among patients aged 18–40 years. Depression (*F* = 3.18, *P* = 0.04), anxiety (*F* = 4.38, *P* = 0.01), and insomnia (*F* = 5.04, *P* = 0.01) were significantly higher in patients with a bachelor's degree or above, compared with other education levels. Those concerned about the economy reported significantly higher depression (*F* = 21.23, *P* < 0.01), anxiety (*F* = 15.64, *P* = 0.01), and insomnia (*F* = 21.92, *P* < 0.01), than those who were not. Significant differences in depression (*F* = 3.27, *P* = 0.01), anxiety (*F* = 2.31, *P* = 0.05) and insomnia (*F* = 2.52, *P* = 0.04) were found among different occupations, and white-collar employees had the worst mental health status.

**Table 1:** Demographic characteristics of patients.

	***n*** **(% of total)**	**Depression**	***F***	**Anxiety**	***F***	**Insomnia**	***F***
Overall	1,014	6.71 ± 5.91		4.81 ± 5.34		8.75 ± 6.13	
**Gender**							
Male	594 (58.6)	6.16 ± 5.83	12.39^**^	4.31 ± 5.21	12.94^**^	8.35 ± 6.30	6.08^*^
Female	420 (41.4)	7.48 ± 5.95		5.52 ± 5.44		9.31 ± 5.83	
**Age**							
<18	35 (3.5)	4.86 ± 4.91	5.14^**^	2.69 ± 3.73	4.71^**^	7.17 ± 5.15	7.79^*^
18–40	584 (57.6)	7.29 ± 5.87		5.27 ± 5.36		9.52 ± 6.12	
40–60	332 (32.7)	6.09 ± 5.93		4.43 ± 5.33		7.81 ± 6.02	
>60	63 (6.2)	5.54 ± 6.09		3.76 ± 5.51		7.30 ± 6.26	
**Education**							
High school or below	414 (40.8)	6.29 ± 6.06	3.18^*^	4.63 ± 5.30	4.38^**^	8.06 ± 6.29	5.04^**^
Junior college	403 (39.7)	6.71 ± 5.80		4.51 ± 5.23		9.03 ± 6.05	
Bachelor or above	197 (19.4)	7.57 ± 5.75		5.81 ± 5.34		9.62 ± 5.81	
**Economic worry**							
Yes	521 (51.4)	7.53 ± 5.94	21.23^**^	5.45 ± 5.46	15.64^**^	9.61 ± 6.07	21.92^**^
No	493 (48.6)	5.84 ± 5.76		4.14 ± 5.13		7.83 ± 6.07	
**Occupation**							
Industrial workers	252 (24.9)	5.83 ± 5.22	3.27^**^	4.08 ± 4.74	2.31^*^	8.20 ± 5.53	2.52^*^
Retirees	93 (9.2)	6.19 ± 6.15		4.69 ± 5.33		7.91 ± 6.31	
Students	55 (5.4)	6.65 ± 6.09		4.71 ± 5.12		8.09 ± 5.58	
Unemployed & others	276 (27.2)	6.68 ± 6.20		4.81 ± 5.34		8.69 ± 6.39	
White-collar employees	338 (33.3)	7.54 ± 5.97		5.42 ± 5.63		9.54 ± 6.32	

* *p* < 0.05;

** *p* < 0.01.

Table 2 describes the correlations of anxiety, depression, and insomnia with each dimension of dysfunctional beliefs about sleep. Results show strong, positive correlations exist between anxiety, depression, and insomnia, and these three factors are all significantly correlated with all of the dimensions of dysfunctional beliefs about sleep to different degrees.

**Table 2:** Pearson correlation analysis among all variables.

	**1**	**2**	**3**	**4**	**5**	**6**
1. Sleep expectation						
2. Assignment of drug	0.44^**^					
3. Consequences of insomnia	0.74^**^	0.69^**^				
4. Worry about sleep	0.65^**^	0.77^**^	0.85^**^			
5. Depression	0.28^**^	0.42^**^	0.42^**^	0.50^**^		
6. Anxiety	0.28^**^	0.42^**^	0.42^**^	0.49^**^	0.86^**^	
7. Insomnia	0.34^**^	0.44^**^	0.46^**^	0.55^**^	0.74^**^	0.69^**^

** *p* < 0.01.

The screening criteria for depression, anxiety, and insomnia were set at PHQ-9 >10, GAD-7 >5, and ISI >8 respectively. Results showed 27.0, 55.3, and 47.8% of the participants have various degrees of depression, anxiety, and insomnia; and 11.3, 17.2, and 16.8% of the total reported moderate or severe depression, anxiety, and insomnia, respectively. Logistic regression analysis showed that female participants were more likely to report depression (OR = 1.35, *P* = 0.04), anxiety (OR = 1.49, *P* < 0.01), and insomnia (OR = 1.48, *P* < 0.01), compared with male participants. Participants who were concerned about the economy were more likely to report depression (OR = 1.97, *P* < 0.01), anxiety (OR = 1.79, *P* < 0.01), and insomnia (OR = 1.67, *P* < 0.01), compared with those who were not. Education, age, and occupation significantly predicted anxiety and insomnia. Participants with a bachelor's degree or above, aged 18–40, and who had worked at a company were more likely to report anxiety and insomnia, see Table 3.

**Table 3:** Prevalence of depression, anxiety and insomnia according to the demographic variables.

	**No. of cases (%)**	**Adjusted OR (95% CI)**	***P***		**No. of cases (%)**	**Adjusted OR (95% CI)**	***P***		**No. of cases (%)**	**Adjusted OR (95% CI)**	***P***
Depression	274 (27.0)			**Anxiety**	453 (44.7)			**Insomnia**	529 (52.2)		
**Gender**				**Gender**				**Gender**			
Male	146 (24.6)	1 [Reference]		Male	146 (24.6)	1 [Reference]		Male	286 (48.1)	1 [Reference]	
Female	128 (30.5)	1.35 (1.02–1.78)	0.04	Female	212 (50.5)	1.49 (1.16–1.92)	< 0.01	Female	243 (57.9)	1.48 (1.14–1.90)	<0.01
**Age**				**Age**				**Age**			
<18	5 (14.3)	1 [Reference]		<18	11 (31.4)	1 [Reference]		<18	14 (40.0)	1 [Reference]	
18–40	175 (30.0)	2.57 (0.98–6.73)	0.06	18–40	290 (49.7)	2.15 (1.03–4.47)	0.04	18–40	338(57.9)	1.03 (1.03–4.13)	0.04
40–60	82 (24.7)	1.97 (0.74–5.24)	0.18	40–60	133 (40.1)	1.49 (0.69–3.08)	0.32	40–60	152 (45.8)	1.27 (0.62–2.58)	0.51
>60	12 (19.0)	1.41 (0.45–4.34)	0.55	>60	19 (30.2)	0.94 (0.39–2.30)	0.90	>60	25 (39.7)	0.99 (0.42–2.30)	0.97
**Education**				**Education**				**Education**			
HSB	105 (25.4)	1 [Reference]		HSB	177 (42.8)	1 [Reference]		HSB	189 (45.7)	1 [Reference]	
JC	112 (27.8)	1.13 (0.83–1.55)	0.43	JC	169 (41.9)	0.97 (0.73–1.28)	0.83	JC	228 (56.6)	1.55 (1.17–2.04)	<0.01
BA	57 (28.9)	1.20 (0.82–1.75)	0.35	BA	107 (54.3)	1.59 (1.13–2.24)	0.01	BA	112 (56.9)	1.57 (1.11–2.21)	0.01
**EW**				**EW**				**EW**			
Yes	174 (33.4)	1 [Reference]		Yes	269 (51.6)	1 [Reference]		Yes	304 (58.3)	1 [Reference]	
No	100 (20.3)	0.51 (0.38–0.68)	<0.01	No	184 (37.3)	0.56 (0.43–0.71)	<0.01	No	225 (45.6)	0.60 (0.47–0.77)	<0.01
**Occupation**				**Occupation**				**Occupation**			
IW	61 (24.2)	1 [Reference]		IW	101 (40.1)	1 [Reference]		IW	129 (51.2)	1 [Reference]	
Retirees	20 (21.5)	0.86 (0.48–1.52)	0.18	Retirees	41 (44.1)	1.18 (0.73–1.91)	0.50	Retirees	46 (49.5)	1.01 (0.64–1.65)	0.91
Students	13 (23.6)	0.97 (0.49–1.92)	0.25	Students	25 (45.5)	1.25 (0.69–2.24)	0.46	Students	25 (45.5)	0.87 (0.49–1.57)	0.65
UE & others	78 (28.3)	1.23 (0.84–1.82)	0.13	LP	119 (43.1)	1.13 (0.80–1.60)	0.48	LP	141 (51.1)	1.10 (0.78–1.54)	0.60
WCE	102 (30.2)	1.35 (0.93–1.96)	0.11	WCE	167 (49.4)	1.46 (1.05–2.03)	0.03	WCE	194 (57.4)	1.41 (1.02–1.96)	0.04

HSB, High school or below; JC, Junior college; BA, Bachelor or above; IW, Industrial workers; UE, Unemployed; WCE, White-collar employees.

Statistical analyses showed 51.4% of the participants had false beliefs about sleep. Table 4 summarizes the prevalence of depression, anxiety, and insomnia according to beliefs about sleep among patients with COVID-19 who were treated in Fangcang shelter hospitals. Accurate beliefs about sleep protect respondents from experiencing depression (χ^2^ = 107.31, *P* < 0.01), anxiety (χ^2^ = 180.30, *P* < 0.01), and insomnia (χ^2^ = 168.48, *P* < 0.01).

**Table 4:** Prevalence of depression, anxiety and insomnia according to respondents' BAS.

**Variable**	**Accurate BAS N (%)**	**False BAS N (%)**	**χ^2^**	***p***
**Depression**				
Yes	60 (12.2)	214 (41.1)	107.31	<0.01
No	433 (87.8)	307 (58.9)		
**Anxiety**				
Yes	114 (23.1)	339 (65.1)	180.30	<0.01
No	379 (76.9)	182 (34.9)		
**Insomnia**				
Yes	154 (31.2)	375 (72.0)	168.48	<0.01
No	339 (68.8)	146 (28.0)		

BAS, beliefs about sleep.

## Discussion

In the current study, we investigated the anxiety, depression, and insomnia among patients treated in Fangcang shelter hospitals during the wave of the COVID-19 pandemic that was dominated by the Omicron variant. The effects of dysfunctional beliefs about sleep on the insomnia, depression, and anxiety of patients in Fangcang shelter hospitals were also explored.

The positive screening rates for anxiety, depression, and insomnia among the tested patients were 55.3, 27.0, and 47.8%, respectively. Amid the pandemic, the positive screening rates of anxiety, depression, and insomnia among the general Chinese adult population were 35.1, 20.1, and 18.2% respectively (25). Of the participants, 11.3, 17.2, and 16.8% reported moderate or severe depression, anxiety, and insomnia. Gu et al. reported 50.1, 54.4, and 10.2% of patients in Fangcang shelter hospitals had moderate or severe symptoms of anxiety, depression, and insomnia, respectively, in 2020 (4), showing significant less participants have moderate or severe affective disorders in our study, compared with patients at the early stages of the pandemic. On the one hand, although the virulence of the Omicron variant is significantly weaker, the proportions of affective and sleep disorders among patients treated in Fangcang shelter hospitals were still higher than those of healthy people. On the other hand, although many patients reported depression, anxiety, or sleep problems to different degrees, the majority of them were mild cases, and the incidence of moderate or severe disorders was significantly reduced compared with patients at the early stages of the pandemic. These facts indicate that attention should be paid to the mental health problems of patients in Fangcang shelter hospitals.

Analysis of variance, and logistic regression analysis of anxiety, depression, and insomnia all demonstrated that patients who were female, aged 18–40 years, had a bachelor's degree or above, were white-collar employees, or those who thought the pandemic would have severe economic effects, reported higher anxiety, depression, and insomnia, and were more likely to report affective and sleep disorders. In particular, those with high education levels were more prone to affective and sleep disorders, which was the opposite result to that of a survey of patients treated in Fangcang shelter hospitals of Wuhan in 2020, conducted by Gu et al., who found patients with lower education levels were more likely to suffer anxiety and depression (4). Possible explanations for this were that during the early pandemic, patients were more worried about their illness status, probable fatality rates, and sequellae (26, 27). As patients with higher education levels had more channels to acquire correct information about the disease, they felt less anxiety or tension (28). Then, 2 years after the pandemic, especially when the low virulence and fatality rates of the Omicron variant were widely reported, the majority of patients were well acquainted with COVID-19 (19). On the contrary, patients with higher education levels were more worried about the sociometric impacts of the pandemic. Our study showed that the proportion of patients with higher education levels who thought the pandemic would have severe economic impacts was significantly higher than that of patients with lower education levels (42.8% of participants with a high school education reported they were very worried about the economy; and the rates were 55.1 and 62.9% for participants in the current study with a junior college education and with bachelors' degree or above, respectively). Thus, patients with higher education levels reported higher anxiety, depression, and insomnia.

Our study showed the dimensions of dysfunctional beliefs about sleep were all significantly positively correlated with insomnia, anxiety, and depression. About 51.4% of participants had dysfunctional beliefs about sleep to varying degrees. Compared to patients with accurate beliefs about sleep, the ratios of insomnia, anxiety, and depression were significantly higher among patients with dysfunctional beliefs about sleep. This result was consistent with a study on Moroccan adults during the pandemic (15). Dysfunctional beliefs about sleep (e.g., unreasonable expectations of the duration of sleep and over-estimation of possible impacts of insomnia) will affect sleep execution (29). The cognitive model of sleep states that dysfunctional beliefs about sleep will drive individuals to conduct some sleep-related protective behaviors, which may induce secondary insomnia. Because of persistent insomnia, their self-feedback about sleep quality and insomnia will promote individuals to strengthen their personal dysfunctional beliefs (30). Patients in Fangcang shelter hospitals may have low-quality sleep due to the unfavorable living environment, and if they hold some dysfunctional beliefs about sleep, such as “I must sleep for a certain length of time so as to maintain energy”; such unrealistic expectations will arouse negative emotions (15). In particular, the worry and sense of helplessness related to insomnia and nightmares will easily arouse anxiety and depression, which may cause difficulty falling asleep, or nocturnal awakening (31–33). At the same time, sleep disorders are significantly and positively correlated with affective disorders (anxiety, depression). This has been extensively shown in many previous studies, as well as in some recent studies during the COVID-19 pandemic (14, 34, 35). So, accurate beliefs about sleep can prevent patients in Fangcang shelter hospitals from depression, anxiety, and insomnia.

Given that dysfunctional beliefs about sleep play a key role in inducing and maintaining insomnia and negative emotions, our findings may imply that we can improve the sleep and mental health condition of patients in Fangcang shelter hospitals by altering their dysfunctional beliefs about sleep. For instance, Edinger et al. thought the dysfunctional beliefs about sleep and attitudes of individuals could be altered by cognitive behavioral therapy (12). It was found that scores of dysfunctional beliefs and attitudes were significantly lowered after the intervention, and participants' sleep quality indices were also largely improved, including: time taken to fall asleep, subjective sleep quality, duration of sleep, and times of awakening at night (12, 36). Thus, we suggest that COVID-19 patients, especially those in Fangcang shelter hospitals, should be kept apprised of correct sleep information and be helped to form correct beliefs about sleep, which, critically, may help maintain their mental health.

This study has several limitations. First, convenience sampling was used, and moderate and severe patients were not investigated. Additionally, all data were collected only in two Fangcang shelter hospitals, so it is impossible to make any inferences about larger populations of COVID-19 patients. Second, due to the time and workload pressure, we failed to collect data on healthy adults for this period of time as a comparable control group. Third, this is a cross-sectional study, and we cannot determine any causal relationships among the variables.

## Conclusions and perspectives

Attention should be paid to the mental health problems of patients in Fangcang shelter hospitals. We recommend giving patients in Fangcang shelter hospitals more psychological support, and helping them to form correct beliefs about sleep, which may help maintain their mental health.

## Data availability statement

The raw data supporting the conclusions of this article will be made available by the authors, without undue reservation.

## Ethics statement

This study was approved by the Clinical Trial Ethics Committee of the First Affiliated Hospital of Air Force Medical University and is registered at the Chinese Clinical Trial Registry, with the registration number CHiCTR1800019761. The patients/participants provided their written informed consent to participate in this study.

## Author contributions

JP and YL were responsible for the writing of this manuscript. LW, XP, CL, XL, JY, and LM were responsible for the data collection. JP and PF were responsible for the data analysis. PF and JS were responsible for the experimental design. TZ was responsible for the revision. All authors approved the final manuscript.

## References

[1] Wang X Fang J Zhu Y Chen L Ding F Zhou R. Clinical characteristics of non-critically ill patients with novel coronavirus infection (COVID-19) in a Fangcang Hospital. Clin Microbiol Infect. (2020) 26:1063–8. 10.1016/j.cmi.2020.03.032 32251842 PMC7195539

[2] Yuan Y Qiu T Wang T Zhou J Ma Y Liu X. The application of Temporary Ark Hospitals in controlling COVID-19 spread: the experiences of one Temporary Ark Hospital, Wuhan, China. J Med Virol. (2020) 92:2019–26. 10.1002/jmv.25947 32343419 PMC7267641

[3] Dai LL Wang X Jiang TC Li PF Wang Y Wu SJ. (2020). Anxiety and depressive symptoms among COVID-19 patients in Jianghan Fangcang Shelter Hospital in Wuhan, China. PloS ONE. 15, e0238416. 10.1371/journal.pone.0238416 32857826 PMC7454940

[4] Gu Y Zhu Y Xu F Xi J Xu G. Factors associated with mental health outcomes among patients with COVID-19 treated in the Fangcang shelter hospital in China. Asia-Pacific Psychiatry. (2021) 13:e12443. 10.1111/appy.12443 33135397

[5] Gu Y Zhu Y Xu G. Factors associated with mental health outcomes among health care workers in the Fangcang shelter hospital in China. Int J Soc Psychiatry. (2022) 68:64–72. 10.1177/0020764020975805 33295238

[6] Zhang GY Liu Q Lin JY Yan L Shen L Si TM. (2021). Mental health outcomes among patients from Fangcang shelter hospitals exposed to coronavirus disease 2019: an observational cross-sectional study. Chronic Dis Transl Med. 7, 57–64. 10.1016/j.cdtm.2020.12.001 33318879 PMC7723755

[7] Brandão LEM Martikainen T Merikanto I Holzinger B Morin CM Espie CA. Social jetlag changes during the COVID-19 pandemic as a predictor of insomnia–a multi-national survey study. Nat Sci Sleep. (2021) 1711–22. 10.2147/NSS.S327365 34675720 PMC8502537

[8] Cénat JM Blais-Rochette C Kokou-Kpolou CK Noorishad PG Mukunzi JN McIntee SE. (2021). Prevalence of symptoms of depression, anxiety, insomnia, posttraumatic stress disorder, and psychological distress among populations affected by the COVID-19 pandemic: a systematic review and meta-analysis. Psychiatry Res. 295, 113599. 10.1016/j.psychres.2020.113599 33285346 PMC7689353

[9] Jahrami H BaHammam AS Bragazzi NL Saif Z Faris M Vitiello MV. (2021). Sleep problems during the COVID-19 pandemic by population: a systematic review and meta-analysis. J Clin Sleep Med. 17, 299–313. 10.5664/jcsm.8930 33108269 PMC7853219

[10] Stanton R To QG Khalesi S Williams SL Alley SJ Thwaite TL. Depression, anxiety and stress during COVID-19: associations with changes in physical activity, sleep, tobacco and alcohol use in Australian adults. Int J Environ Res Public Health. (2020) 17:4065. 10.3390/ijerph17114065 32517294 PMC7312903

[11] Lin W Wu B Chen B Lai G Huang S Li S. Sleep conditions associate with anxiety and depression symptoms among pregnant women during the epidemic of COVID-19 in Shenzhen. J Affect Disord. (2021) 281:567–73. 10.1016/j.jad.2020.11.114 33261931 PMC7688420

[12] Edinger JD Wohlgemuth WK Radtke RA Marsh GR Quillian RE. Does cognitive-behavioral insomnia therapy alter dysfunctional beliefs about sleep? Sleep. (2001) 24:591–9. 10.1093/sleep/24.5.591 11480656

[13] Morin CM Vallières A Ivers H. Dysfunctional beliefs and attitudes about sleep (DBAS): validation of a brief version (DBAS-16). Sleep. (2007) 30:1547–54. 10.1093/sleep/30.11.1547 18041487 PMC2082102

[14] Blake MJ Trinder JA Allen NB. Mechanisms underlying the association between insomnia, anxiety, and depression in adolescence: implications for behavioral sleep interventions. Clin Psychol Rev. (2018) 63:25–40. 10.1016/j.cpr.2018.05.006 29879564

[15] Idrissi AJ Lamkaddem A Benouajjit A El Bouaazzaoui MB El Houari F Alami M. (2020). Sleep quality and mental health in the context of COVID-19 pandemic and lockdown in Morocco. Sleep Med. 74, 248–253. 10.1016/j.sleep.2020.07.045 32862008 PMC7422815

[16] Sella E Carbone E Toffalini E Borella E. Self-reported sleep quality and dysfunctional sleep-related beliefs in young and older adults: changes in times of COVID-19 lockdown. Sleep Med. (2021) 81:127–35. 10.1016/j.sleep.2021.02.017 33676283 PMC9182325

[17] Bacaro V Chiabudini M Buonanno C De Bartolo P Riemann D Mancini F. (2020). Insomnia in the Italian population during Covid-19 outbreak: a snapshot on one major risk factor for depression and anxiety. Front Psychiatry 11, 579107. 10.3389/fpsyt.2020.579107 33384625 PMC7769843

[18] Xu Z Lu H Zhou J Feng X Hambly B Fan J. Implementation of emergency general practitioner management of patients during the complete lockdown consequent to the COVID-19 Omicron outbreak in Shanghai. Hypertension. (2022) 1:0–0001. 10.31128/AJGP-COVID-51-5 35790217

[19] Brüssow H. COVID-19: Omicron–the latest, the least virulent, but probably not the last variant of concern of SARS-CoV-2. Microb Biotechnol. (2022) 15:1927–39. 10.1111/1751-7915.14064 35443078 PMC9111164

[20] Fu S Ou H Lu S. Reliability and validity of the brief version of dysfunctional beliefs and attitudes about sleep. Chin J Behav Med Brain Sci. (2014) 23:369–71. 10.3760/cma.j.issn.1674-6554.2014.04.024

[21] Spitzer RL Kroenke K Williams JBW Löwe B. A brief measure for assessing generalized anxiety disorder: the GAD-7. Arch Intern Med. (2006) 166:1092–7. 10.1001/archinte.166.10.1092 16717171

[22] Ruiz MA Zamorano E García-Campayo J Pardo A Freire O Rejas J. Validity of the GAD-7 scale as an outcome measure of disability in patients with generalized anxiety disorders in primary care. J Affect Disord. (2011) 128:277–86. 10.1016/j.jad.2010.07.010 20692043

[23] Manea L Gilbody S McMillan D. A diagnostic meta-analysis of the Patient Health Questionnaire-9 (PHQ-9) algorithm scoring method as a screen for depression. Gen Hosp Psychiatry. (2015) 37:67–75. 10.1016/j.genhosppsych.2014.09.009 25439733

[24] Bastien CH Vallières A Morin CM. Validation of the Insomnia Severity Index as an outcome measure for insomnia research. Sleep Med. (2001) 2:297–307. 10.1016/S1389-9457(00)00065-4 11438246

[25] Galea S Merchant RM Lurie N. The mental health consequences of COVID-19 and physical distancing: the need for prevention and early intervention. JAMA Intern Med. (2020) 180:817–8. 10.1001/jamainternmed.2020.1562 32275292

[26] Kavakli M Ak M Uguz F Türkmen OO. The mediating role of self-compassion in the relationship between perceived COVID-19 threat and death anxiety. J Clin Psychiatry. (2020) 23:15–23. 10.5505/kpd.2020.59862

[27] Özgüç S Kaplan Serin E Tanriverdi D. Death anxiety associated with coronavirus (COVID-19) disease: a systematic review and meta-analysis. OMEGA J Death Dying. (2021) 8:00302228211050503. 10.1177/00302228211050503 34622711 PMC10768329

[28] Deng J Zhou F Hou W Silver Z Wong CY Chang O. (2021). The prevalence of depressive symptoms, anxiety symptoms and sleep disturbance in higher education students during the COVID-19 pandemic: a systematic review and meta-analysis. Psychiatry Res. 301, 113863. 10.1016/j.psychres.2021.113863 33984824 PMC9225824

[29] Garland SN Campbell T Samuels C Carlson LE. Dispositional mindfulness, insomnia, sleep quality and dysfunctional sleep beliefs in post-treatment cancer patients. Pers Individ Dif. (2013) 55:306–11. 10.1016/j.paid.2013.03.003

[30] Harvey AG Tang NKY Browning L. Cognitive approaches to insomnia. Clin Psychol Rev. (2005) 25:593–611. 10.1016/j.cpr.2005.04.005 15979771

[31] Calkins AW Hearon BA Capozzoli MC Otto MW. Psychosocial predictors of sleep dysfunction: the role of anxiety sensitivity, dysfunctional beliefs, and neuroticism. Behav Sleep Med. (2013) 11:133–43. 10.1080/15402002.2011.643968 23136825

[32] Jin L Zhou J Peng H Ding S Yuan H. Investigation on dysfunctional beliefs and attitudes about sleep in Chinese college students. Neuropsychiatr Dis Treat. (2018) 14:1425–32. 10.2147/NDT.S155722 29910619 PMC5989819

[33] Scarpelli S Nadorff MR Bjorvatn B Chung F Dauvilliers Y Espie CA. Nightmares in people with COVID-19: did coronavirus infect our dreams? Nat Sci Sleep. (2022) 14:93. 10.2147/NSS.S344299 35115852 PMC8800372

[34] Johnson EO Roth T Breslau N. The association of insomnia with anxiety disorders and depression: exploration of the direction of risk. J Psychiatr Res. (2006) 40:700–8. 10.1016/j.jpsychires.2006.07.008 16978649

[35] Morin CM Bjorvatn B Chung F Holzinger B Partinen M Penzel T. (2021). Insomnia, anxiety, and depression during the COVID-19 pandemic: an international collaborative study. Sleep Med. 87, 38–45. 10.1016/j.sleep.2021.07.035 34508986 PMC8425785

[36] Thakral M Von Korff M McCurry SM Morin CM Vitiello MV. Changes in dysfunctional beliefs about sleep after cognitive behavioral therapy for insomnia: a systematic literature review and meta-analysis. Sleep Med Rev. (2020) 49:101230. 10.1016/j.smrv.2019.101230 31816582 PMC7012685

